# Exit-Knowledge of Ambulatory Patients About Medications Dispensed in Government Hospital in Eastern Ethiopia: The Need for Focused Patient Counseling

**DOI:** 10.3389/fpubh.2018.00254

**Published:** 2018-09-05

**Authors:** Nigatu Hirko, Dumessa Edessa, Mekonnen Sisay

**Affiliations:** ^1^Department of Pharmacy, Bisidimo General Hospital, Oromia, Ethiopia; ^2^Department of Clinical Pharmacy, School of Pharmacy, College of Health and Medical Sciences, Haramaya University, Harar, Ethiopia; ^3^Department of Pharmacology and Toxicology, School of Pharmacy, College of Health and Medical Sciences, Haramaya University, Harar, Ethiopia

**Keywords:** dispensed medications, patient, exit-knowledge, outpatient pharmacy, Federal Harar Police Hospital

## Abstract

**Background:** In the counseling process, a multi-cultural competence of dispenser is among the key factors affecting his/her successful communication with patients for achieving optimal use of medications. For patients to use dispensed drugs appropriately, it is a must for them to understand the medication related information provided by the dispenser. Hence, the objective of this study was to identify parameters that likely affect ambulatory patients' knowledge of medication(s) provided at the exit of outpatient pharmacy of Federal Harar Police Hospital, Eastern Ethiopia.

**Methods:** Cross-sectional study design was employed to conduct this study. An interview of patients was made at the exit of hospital pharmacy using a semi-structured questionnaire. The interview tool primarily assessed the knowledge of the patients for a maximum of three medications provided. Statistical Package for Social Sciences (SPSS), Version 20.0, was employed for analysis of the data. Chi-squared test was done to retain parameters with potential to have association; and the retained parameters were adjusted by performing bivariate and multivariate logistic regression analyses.

**Results:** The result showed that only 37.2, 33.4 and 28.7% of the patients were able to recall the name of the drug(s), common side effects, and actions to be taken for missed doses, respectively. The likelihood of patients' knowledge for dispensed medications was high among patients aged 19–39 years (adjusted odds ratio [AOR]: 5.0; 95% CI: 1.04–24.2) and who thought their communication with dispenser had been polite (AOR: 4.62; 95% CI: 1.48–14.4). However, the knowledge status was found low among patients who were Afan Oromo speakers (AOR: 0.58; 95% CI: 0.35–0.95) and who came from rural residence (AOR: 0.48; 95% CI: 0.25–0.90).

**Conclusion:** A high proportion of patients were unable to recall the drug (s) name, associated common toxicities, and actions to be taken in case of missed dose. In addition, patients who were at early adulthood and who were positive for the politeness of dispenser had better exit-knowledge of their medication. Therefore, for the patients' clear understanding of medications provided, it is mandatory to optimize patient–dispenser communication possibly by adapting multi-cultural communication skills and by providing focused training for dispensers to address factors that likely affect patient-dispenser interactions.

## Introduction

Drug dispensing is a process that ends when a patient leaves drug retail outlets with a defined quantity of medication(s) and adequate instruction (s) provided on how to use them (1). It is one of the focal areas that drug use can be promoted in healthcare system. The way in which drugs are used by the patients is commonly affected by the overall protocol of dispensers and information delivered by them during the dispensing process. The time that the dispenser spends with patients during drug use process is referred to as dispensing or counseling time which is an important indicator of the quality of service delivery (2–4); and this has to be critically considered with regard to the dispenser's role and competence in effective patient counseling.

Pharmacists should be engaged in optimizing patient safety throughout the medication use processes. The role of the dispensers have been proven to improve several health outcomes including greater patient safety, better disease and drug therapy management, more efficient healthcare expenditure, higher adherence status, and better quality of life (5). Therefore, patient counseling by dispenser is a key competency element in the medication treatment process. To this end, it is critical for the dispensers to provide desirable and understandable information to patients about their dispensed medications. The dispenser is in a critical position to answer whatever concerns and enquiries of patients toward their medications and even alternate therapeutic approaches they may seek or hear from others. Counseling should include an evaluation of whether the information was received as required and that the patient comprehends how to use the information he/she receives to improve positive therapeutic outcomes. Appropriate means of delivering information to patients is also essential to encourage effective and safe use of medication(s) by them. Accordingly, the Australian Pharmaceutical Advisory Council outlines the type of information and resources that should be delivered to patients. Moreover, list of medicines provided on exit from the healthcare facility should be prepared in communication and collaboration with the patient for ease of improving adherence to treatment regimens and patient outcomes (6–9).

Communication failure between patients and healthcare providers can lead to gaps in the continuity of the treatment process. Health professionals are prescribing and dispensing a large number of medications without providing sufficient information to the patients. To this end, studies showed that higher workload of dispensers, defined as the number of prescriptions dispensed per dispenser working hour led to increased risk of dispensing a potentially unsafe medication and unlikely to provide thorough patient counseling about their dispensed medications (6, 10). In addition, certain patients are potentially vulnerable to medication errors. These include patients with specific conditions (e.g., pregnancy, renal, and hepatic dysfunctions); patients taking multiple medications prescribed by the same or different professionals; patients with multiple health problems; patients who are passive about their own health; patients with dementia (memory problems) and patients who have communication difficulty, young children, and patients who do not speak the same language as the dispenser, are particularly vulnerable to medication errors (6, 11). Studies that assess the exit-knowledge status of patients toward their dispensed medication at outpatient setting are very limited in Ethiopia. Therefore, this study aimed to identify dispenser and patient related factors that likely affect the knowledge of ambulatory patients toward medication dispensed at outpatient pharmacy of Federal Harar Police Hospital (FHPH).

## Methods

### Study area, design, and period

This study was conducted at FHPH outpatient pharmacy. The FHPH is one of the government hospitals found in Harar town located 526 km away from the capital of Ethiopia, Addis Ababa to the east. An institution-based cross-sectional study design was employed to assess the knowledge status of patients served at the outpatient pharmacy of the hospital from February to April, 2016.

### Study population and inclusion/exclusion criteria

Ambulatory patients who visited the pharmacy unit of FHPH to receive medications dispensed to them during the data collection period were included for the study. Those who were seriously ill and/or who could not give their consent to participate in the study were excluded. In addition, due to concerns pertaining to patients' retention and recall capability of information in case of high number of dispensed drugs, those who received four and more drugs from the pharmacy were not considered for the interview.

### Sample size determination and sampling technique

The number of study participants was determined by using single population proportion formula (${\text{Z}}_{\text{α}}^{2}$pq/d^2^). For this, alpha (α) value of 0.05, confidence level (Cl) of 95% and proportion (p) of 0.50 for knowledge status of patients at the exit of outpatient pharmacy, and 5% margin of error (d) were employed. Accordingly, 384 patients were obtained as the exact sample size of study population was not known in such prospective study and this number was finally adjusted to 422 patients by adding 10% for certain methodological non-contingency. During the time of data acquisition, convenient sampling technique was employed to enroll study participants for interview.

### Study variables

Socio-demographic parameters such as age, sex, area of residence, educational status, ethnicity, and primary language of communication along with patient-dispenser communication parameters were considered as explanatory variables. In addition, patient's level of exit-knowledge about their dispensed medication was referred as a key outcome variable. A patient was assumed to be with adequate exit-knowledge about the medication(s) dispensed when he/she definitely addressed two-thirds and more of the necessary knowledge related questions.

### Data collection tools and methods

A customized semi-structured data collection tool was employed for interviewing the patients during exit from the hospital pharmacy. Good dispensing practice variables adopted by the World Health Organization (WHO) and the Ethiopian Food, Medicine and Healthcare Administration and Control Authority (FMHACA) were considered partly for the preparation of the questionnaire (12, 13). This tool consisted of patient related characteristics including age, sex, place of residence, educational level, occupation, marital status, and type of language spoken, along with patient-dispensary communication and interaction parameters that likely affect the knowledge level of how to take dispensed medications. Accordingly, the tool has two sections: the first section contained the socio-demographic and patient-dispenser characteristics; the second part of the tool addressed the knowledge status of patients about the medication(s) dispensed to them at exit of hospital pharmacy (Supplementary Table 1).

### Pretest and quality control

In this study, various techniques were tried to guarantee the quality of the data collected. Before the actual data collection, the drafted interview tool was ultimately enhanced by pre-testing on 5% of patients (22 patients) at dispensary unit of Jugel Hospital which is found in the same town. Following certain amendments based on the pretest data, the data collection tool was employed for the actual data gathering. The data collection was conducted by two pharmacists who had been vividly trained about the purpose and method of data collection. The data collectors handled the interview process while also checking the responses provided immediately after completing every interview. Consistency of the questionnaire was maintained by translating English language into local languages (Afan Oromo and Amharic). These local language contents of the tool were back translated into English to ensure consistency of the translations. At every interview, each patient was also well informed about the goal of the study while simultaneously addressing the importance of replying to all the inquiries sincerely as well. Finally, the completeness of each datum was ensured daily by the investigators and comments were provided to the data collectors accordingly.

### Statistical analysis

The data were coded for suitable entry into Epi-info Version 3.5.1 and exported to Statistical Package for Social Science version 20.0 (IBM Statistics, Armonk NY, United States) for statistical analysis. Socio-demographics characteristics, patient-dispenser communication and interaction parameters, and exit-knowledge level of patients about the dispensed medication(s) were summarized by using frequency and percentages (univariate analyses). Potential variables with *p* < 0.2 by Pearson chi-squared test (χ^2^) were retained for subsequent consideration in binary logistic regression analysis. Finally, variables retained after the χ^2^ test were regressed against the knowledge status of patients. Accordingly, age, place of residence, primary language spoken by the patient, frequency of pharmacy visit within the last 6 months, patient's perception about dispenser's politeness during counseling process, and perceived interaction status between patient and dispenser were the key variables considered for both bivariate and multivariate regression against the outcome variable. Significant association was declared at *p* < 0.05 and 95% confidence level.

### Ethical consideration

Study approval and ethical clearance was sought and received from Haramaya University, College of Health and Medical Sciences, School of Pharmacy. A clearance letter was also obtained from the College of Health and Medical Sciences with reference number C/A/R/D/01/1611/16 for conducting this research in FHPH. Official permission was then received from hospital administrator to start the study. Voluntary, informed, written and signed consent was also obtained from every study participant after the purpose of the study was introduced to him/her prior to conducting actual interview. Confidentiality of the collected data was maintained in such a way that the data collection tool was kept anonymous.

## Results

A total of 422 patients were included in the study. More than half of the patients were in age range between years 19 and 39 (57.8%), were married (70.6%), were Orthodox Christian (48.6%), had finished secondary levels of education (41.2%), belonged to the Oromo (41.9%) and Amhara (37.2%) ethnic groups, were Amharic language speakers (65.9%) and were urban dwellers (83.9%). Similarly, about three-fourth (74.2%) and two-thirds (65.6%) of the patients were more frequent visitors of pharmacy and were comfortable with the waiting facility of the dispensary unit, respectively. Moreover, at least two-thirds of the patients perceived about their interaction with dispensers as promising. Accordingly, the patients thought that their interaction with dispensers was encouraging in terms of ascent of dispensers (87.2%), manner of dispensers (82%), guidance of dispensers (79.4%), and communication of dispensers (68.2%) (Table 1).

**Table 1:** Socio-demographics and perceived communication status of study participants at FHPH, Harar, February-April, 2016.

**Characteristics**	**Frequency (%)**
**Sex**	
Male	215 (50.9)
Female	207 (49.1)
**Age (years)**	
≤ 18	13 (3.1)
19–39	244 (57.8)
40–59	126 (29.9)
≥60	39 (9.2)
**Marital status**	
Single	90 (21.3)
Married	298 (70.6)
Divorced	13 (3.1)
Widowed	19 (4.5)
Separated	2 (0.5)
**Religion**	
Muslim	148 (35.1)
Orthodox	205 (48.6)
Protestant	68 (16.1)
Catholic	1 (0.2)
**Occupation**	
Farmer	5 (1.2)
Government employee	200 (47.4)
Merchant	56 (13.3)
Laborer	14 (3.3)
Private employee	28 (6.6)
Student	43 (10.2)
Housewife	48 (11.4)
Retirement	28 (6.6)
**Educational status**	
Illiterate	25 (5.9)
Can read and write	32 (7.6)
Primary	121 (28.7)
Secondary	174 (41.2)
Tertiary	70 (16.6)
**Ethnicity**	
Oromo	177 (41.9)
Harari	44 (10.9)
Amhara	157 (37.2)
Tigrie	27 (6.4)
Somali	10 (2.4)
Other^*^	7 (1.7)
**Place of residence**	
Urban	354 (83.9)
Rural	68 (16.1)
**Frequency of dispensary unit visit in the last 6 months**	
First time	24 (5.7)
Second time	85 (20.1)
Repeated times	313 (74.2)
**Primary language of communication by patient**	
Amharic	278 (65.9)
Oromo	118 (28.0)
Adarigna	19 (4.5)
Tigirigna	7 (1.7)
**Perceived communication ranked by the patient**	
Poor	30 (7.1)
Fair	104 (24.6)
Good	288 (68.2)
**Perceived ascent status of the dispenser**	
Clear	368 (87.2)
Not clear	54 (12.8)
**Perceived comfort of waiting area**	
Not suitable	73 (17.3)
Fairly suitable	72 (17.1)
Suitable	277 (65.6)
**Perceived politeness of the dispenser**	
Impolite	27 (6.4)
Fairly polite	49 (11.6)
Polite	346 (82.0)
**Perceived clarity of the dispenser's guidance**	
Not clear	39 (9.2)
Fairly clear	48 (11.4)
Clear	335 (79.4)
**Perceived sufficiency of the dispenser' information**	
Not enough	115 (27.3)
Don't know	34 (8.1)
Enough	273 (64.7)

Asterisk (

* *) stands for Gurage, Sidama, and Argoba. FHPH, Federal Harar Police Hospital*.

At exit from the dispensary unit, study patients were assessed whether they had the required information of the medication they received as summarized in Table 2. As per the result, at least more than half of the patients recalled the required information related to their the medication frequency of use (82.9%), route of administration (82.5%), direction of use (72.3%), expected therapeutic outcome (63%), storage conditions (54.9%), and drug interaction (54.7%). Contrary to these, only 37.2, 33.4 and 28.7% of the patients were able to remember name of the medication, potential toxicities, and actions taken in cases of forgotten doses, respectively. Generally, only 38.6% of the patients fulfilled the predefined criteria and hence considered to have adequate knowledge (Table 2).

**Table 2:** Knowledge status of patients about their dispensed drugs at exit of pharmacy of FHPH, February-April, 2016 (*n* = 422).

**Patient's knowledge status (recalling capability)**	**Frequency (%)**
**Name of medication(s) dispensed**	
No	265 (62.8)
Yes	157 (37.2)
**Medication's indication**	
No	155 (36.7)
Yes	267 (63.3)
**Route of administration**	
No	74 (17.5)
Yes	348 (82.5)
**Duration of therapy**	
No	199 (47.2)
Yes	223 (52.8)
**Medication's frequency**	
No	72 (17.1)
Yes	350 (82.9)
**Drug interaction**	
No	191 (45.3)
Yes	231 (54.7)
**Common potential toxicities**	
No	281 (66.6)
Yes	141 (33.4)
**Direction on how to use medication**	
No	117 (27.7)
Yes	305 (72.3)
**Actions taken for forgotten dosage**	
No	301 (71.3)
Yes	121 (28.7)
**Appropriate handling of the received drug**	
No	191 (45.4)
Yes	231 (54.7)
**Medication's label**	
No	138 (32.7)
Yes	284 (67.3)
**Expected therapeutic outcome**	
No	156 (37.0)
Yes	284 (63.0)
**Average sufficiency of the awareness**	
Sufficient (at least two-thirds of correct answers)	163 (38.6)
Not sufficient (less than two-thirds of correct answers)	259 (61.4)

*FHPH, Federal Harar Police Hospital*.

On binary logistic regression analysis, several variables were found to have association with patients' knowledge of how to use their dispensed drugs (Table 3). Accordingly, patients who thought the manner of dispenser as fairly positive (crude odds ratio [COR], 4.31; 95% CI, 1.29–14.36) or positive (COR, 3.81; 95% CI, 1.29–11.27) had more understanding of the drug (s) they received from pharmacy. Multivariate analysis also revealed a similar finding to the above bivariate analysis and patients who thought manner of the dispenser as positive had more odds of understanding for dispensed drug (s) (adjusted odds ratio [AOR], 4.62; 95% CI, 1.48–14.4) compared to patients who thought manner of the dispenser as negative. Furthermore, patients who were in the age range between years 19 and 39 (AOR: 5.0; 95% CI: 1.04–24.2) had more likelihood of understanding of the drug(s) they received compared to patients aged ≤ 18 years. Nevertheless, a significantly reduced exit knowledge about the dispensed drug(s) was noted among patients who spoke Afan Oromo (AOR: 0.58; 95% CI: 0.35–0.95) and who were rural residents (AOR, 0.48; 95% CI, 0.25–0.90) compared to patients who spoke Amharic and who were urban residents, respectively (Table 3).

**Table 3:** Regression analysis for factors associated with knowledge of patients for dispensed drugs at dispensary unit of FHPH, Harar, February-April, 2016.

**Variable**	**COR [95% CI]**	***P*-values**	**AOR [95% CI]**	***P*-values**
**Age (years)**				
≤ 18	1		1	
19–39	4.2 [0.92–19.46]	0.065	5.0 [1.04–24.2]	0.045^*^
40–59	2.2 [0.46–10.42]	0.32	2.24 [0.45–11.1]	0.32
≥60	5.2 [1.02–26.72]	0.047	4.36 [0.84–23.8]	0.08
**Residence**				
Urban	1		1	
Rural	0.43 [0.24–0.80]	0.006	0.48 [0.25–0.90]	0.02^*^
**Primary language the patient spoke**				
Amharic	1		1	
Oromo	0.60 [0.38–0.95]	0.03	0.58 [0.35–0.95]	0.03^*^
Adarigna	1.26 [0.49–3.19]	0.63	1.12 [0.41–3.02]	0.82
Tigirigna	0.93 [0.15–5.66]	0.94	0.89 [0.13–5.97]	0.90
**Perceived politeness of the dispenser**				
Negative	1		1	
Fairly positive	4.31 [1.29–14.36]	0.017	1.71 [0.68–4.32]	0.55
Positive	3.81 [1.29–11.27]	0.015	4.62 [1.48–14.4]	0.008^*^
**Frequency of dispensary visit in the last 6 months**				
First time	1		1	
Second time	2.0 [0.72–5.55]	0.18	2.36 [0.78–7.08]	0.12
Repeated visit	1.94 [0.75–5.03]	0.17	1.70 [0.61–4.71]	0.30
**Perceived interaction of patient with dispenser**				
Poor	1		1	
Fair	1.52 [0.61–3.75]	0.36	1.38 [0.52–3.68]	0.52
Good	1.90 [0.82–4.43]	0.13	1.71 [0.68–4.32]	0.25

Asterisk (

* *) shows statistical significance. CI, confidence interval; FHPH, Federal Harar Police Hospital*.

## Discussion

This study generally included 422 patient attendees in the FHPH during the study period. The overall exit-knowledge of patients can be affected by a multitude of factors. Among which, this study mainly emphasized on patient and dispenser related factors that may possibly affect the exit-knowledge of dispensed drugs. Binary logistic regression was applied to show whether there is a statistically significant association between the exit- knowledge of patients with several predictor variables including residence of patients, age, the type of language they primarily spoke, perceived politeness of the dispensers, frequency of hospital pharmacy visits and the nature of dispenser-patient interactions.

In this study, 89.2, 82.5, 72.3, 63, 54.9, and 54.7% of patients recalled frequency of medication, route of administration, medication instruction, expected therapeutic outcome, proper storage conditions, and drug interactions, respectively. However, less than half of the patients (37.2, 33.4, and 28.7%) were able to recall the name of the medication, the major side effects, and actions taken in cases of missed doses, respectively. Overall, less than half (38.6%) of the ambulatory patients met the pre-defined criteria of exit knowledge about dispensed drugs. In relation with this finding, study conducted at rural Gambia showed that following an interview on exit knowledge of dispensed drugs, 60.4, 5.4, and 17.2% patients correctly recalled the drug dosage, the duration of treatment, and the purpose of treatment, respectively. Overall, 16.1% of patients' responses met the predefined criteria for ‘good’ level of exit knowledge of dispensed drugs (14). Similarly, a research conducted at Jimma University Specialized Hospital showed that the mean patients' exit knowledge score was found to be 3.7 out of 7. Routes of administration (100.0%), dose (96.1%), frequency (95.5%), indication (89.3%), duration of therapy (49.6%), and the name of medicine (15.1%) were recalled among the dispensed drugs (15).

In this study, the multivariate analysis indicated that statistically significant exit knowledge of dispensed drugs was observed in patients whose age was within 19–39 years compared to patients aged ≤18 years [AOR = 5.0; 95% CI: 1.04–24.2]. In agreement to our study, a study conducted in outpatient settings revealed that being adult and older age were associated with higher levels of exit knowledge compared to younger patients (*p* < 0.05) (16). Furthermore, another study indicated that older patients were generally more satisfied with the consultations given by dispensers, having greater exit knowledge of dispensed drugs as well (14, 17). On the other end, a contradictory finding was also reported by Marks et al. (18) in which the medication knowledge score was affected positively by younger age. Besides, a study done by Kerzman et al. (19) has also reported another findings stating that there was no statistically significant effect of age on medication knowledge of patients.

Residence of patients can also affect the exit knowledge of ambulatory patients about their dispensed medication(s). The knowledge of dispensed drugs was significantly decreased among patients who were rural residents compared to those who were urban dwellers. In concordant to the present finding, a study conducted at Shambu primary hospital, southwest Ethiopia indicated that misunderstanding of dosage regimen instructions was significantly associated with residence. Those patients who came from rural residence were more likely to misunderstand instructions compared to urban dwellers (χ^2^ = 13.8, *p* < 0.001) (20).

Regarding the barrier for effective communication between pharmacists and patients in outpatient settings, language barrier is one of the most important but often overlooked barriers in area of clinical settings. In the present study, the knowledge of dispensed drugs was significantly decreased among patients who were Afan Oromo speakers (AOR: 0.58; 95% CI: 0.35–0.95) compared to those patients who were Amharic speakers. There are several literatures that stand for supporting the present finding. Generally, communication has been recognized as a huge potential barrier in healthcare provider- patient interaction and there is evidence of interference from psychosocial, cultural and linguistic barriers. The way and the language in which the healthcare provider communicates and the patient understands are very critical since poor communication may lead to non-adherence to medicines (21). Some patients may show motivation to adhere with the prescribed medications; however, fail to do so because of misunderstanding associated with several factors including low literacy level and language barriers which are essential factors for unintentional non-adherence (22). Language barriers between pharmacist-patient communications may lead to diminished patient satisfaction with treatment; lower understanding of medication counseling; reduced adherence to prescribed medications; fewer follow-up visits; and poorer treatment outcomes. (23–26). Similarly, good communication facilitates the counseling process and results in more appropriate treatment regimens and better patient compliance. Moreover, good communication also benefits the healthcare system as a whole by making it more efficient and cost-effective (27).

Looking at perceived interaction status of patients with pharmacist, in spite of statistically insignificant association, there is an increasing trends of exit knowledge level of ambulatory patients when the perceived interaction become stronger. Accordingly, patients who perceived their interaction as good and fair have 1.71 and 1.38 times more odds of exit knowledge of dispensed drugs, respectively, compared to those who perceived the patient-pharmacist interaction as poor. Supporting this study, another study showed that there was a close correlation between patients' knowledge of dispensed drugs and pharmacist interaction (*r* = 0.95) with the patient. The interaction between dispensers and patients increases exit knowledge of dispensed medicines and it was reported that written directions are very useful when they are supplemented by an oral explanation. The most effective tasks in promoting the pharmacist–patient interaction were obtaining patients' history and provision of verbal instruction. Besides, eye contact was significantly related to patient perceptions of clinician empathy and connectedness (28–30). However, very short dispensing time was considered as a major factor for inadequate provision of medication counseling and hence results in poor exit knowledge of patients about their dispensed drugs (31). On the top of this, dispensers can undergo interactive communication and provision of instructions by actively involving the patients in their own health. Since patients forget more than half of the information delivered to them via oral communication immediately after they hear it, strategies aimed at improving patients' recall of medical instructions must be used to assist patients with their prescribed medications (22). Another study also emphasized that pharmacists still have a way to go to fully address patients' healthcare demands, particularly in culturally diverse settings (32–34). One report indicated that among patients with chronic conditions who came for medication refills, 41.8% believed that, although they needed counseling, it was not provided by the pharmacist (17).

Coming to the perceived politeness of pharmacist, multivariate analyses revealed a statistically significant association between the politeness of pharmacist and patients' knowledge about dispensed drugs. That is, patients who perceived the behavior of pharmacist as polite had 4.62 times more odds of the exit knowledge of dispensed drugs compared to those who perceived the pharmacist behavior as impolite. On this side, there have been some research findings and hypothesis supporting the present study. It is important that dispensers establish a positive, supportive and trusting relationship with the patient. One must adopt a friendly rather than a business-like attitude toward the patients he/she serves (35). Furthermore, the existence of optimum and positive interaction that can be achieved between pharmacists and their patients has been shown to originate from the pharmacists' communication skill and use of motivational conversation (36, 37).

Regarding the frequency of outpatient pharmacy visits, despite statistically insignificant association, patients who had experiences of second or repeated visits to the hospital pharmacy had increased odds of the exit knowledge of dispensed drugs. Consistent to the present finding, previous counseling was positively linked to the medication knowledge (*P* < 0.05). About 87.8–97.6% of patients who received the previous counseling showed good to excellent recognition of medication knowledge of their indications (16).

### Limitation of the study

This study was not without potential limitations. The authors tried to assess the exit knowledge status of ambulatory patients about dispensed drugs. However, the exit knowledge status was highly subjective to the information retention and recall ability of each patient. This subjective nature of the data could result into an underestimation of the knowledge measurement. This measurement, therefore, considered only those patients who received less than or equal to three drugs from the outpatient pharmacy to assess the exit knowledge of the ambulatory patients since drug information retention ability of the patients was thought to be affected by the number of drugs dispensed to them. On top of these limitations, patients who responded at least two-thirds of the knowledge questions were considered knowledgeable for the dispensed drugs, and this might have also resulted into underestimation of the exit knowledge status. Hence, any interpretation of the findings in this study should be done in consideration of the aforementioned limitations.

## Conclusion

The present study found that less than half of the patients met the defined criteria for adequate exit knowledge and various factors related to patient and dispenser affected the exit knowledge of patients on how to use dispensed drugs. The exit knowledge was significantly increased among patients at early adulthood. Perceived politeness of dispensers by patients was also found to have statistically significant association with the exit knowledge. However, the exit knowledge was significantly decreased among patients who were rural residents and were Afan Oromo speakers. Therefore, increasing educational coverage and standards targeting the rural community and improving multi-cultural communication skill based trainings for dispensers will likely optimize patient–dispenser communication, which in turn will improve the exit knowledge of patients about medications dispensed to them.

## Author contributions

NH has conceived the study, participated in study design, data acquisition, and interpretation of results. DE has also conceived the study, participated in the study design, data acquisition, statistical analysis, and interpretation of results. MS has also participated in the study design, data acquisition, analysis, and interpretation of findings, and manuscript drafting and writing. The three authors have participated sufficiently in contributing to the writing of the manuscript and its final approval.

### Conflict of interest statement

The authors declare that the research was conducted in the absence of any commercial or financial relationships that could be construed as a potential conflict of interest.

## References

[1] WHO Introduction to Drug Utilization/WHO International Working Group for Drugs Statistics Methodology. Oslo: WHO collaborating centers for drug statistic and methodology (2003).

[2] Agency for Health Care Research and Quality (AHCQ) One Third of Elderly Home Health Care Patients are Having Problems With Their Medication (2003).

[3] Margaret-Mary A David OA The impact of three forms of educational interventions on dispensing practices. In: International Conferences on Improving Use of Medicines (ICIUM) (Chang Mai) (1997).

[4] WHO How to Investigate Drug Use in Health Facilities: Selected Drug Indicators Action Program on Essential Drugs (DAP). Geneva: WHO (1993). p. 9–31.

[5] Shah A Pharmacy Intervention in the Medication Use Process, The Role of Pharmacists in Improving Patient Safety. Winnipeg, MB: University of Manitoba (2010). p. 1–50.

[6] WHO WHO Patient Safety Curriculum Guide for Medical Schools. Geneva: WHO (2009). p. 242–3.

[7] Australian Pharmaceutical Advisory Council (APAC) Guiding Principles to Achieve Continuity in Medication Management. Camberra, ACT: Commonwealth of Australia (2005). p. 1–52.

[8] WHO Promoting Rational Use of Medicines: Core Components. Geneva: WHO (2002). p. 1–6.

[9] Utilization Review Accreditation Commission (URAC) Supporting Patient Medication Adherence: Ensuring Coordination, Quality and Outcomes. Columbia, WA: URAC (2011). p. 1–22.

[10] Nair RP Kappil D Woods TM 10 strategies for minimizing dispensing errors. Pharmacy Times (Accessed January 20, 2010).

[11] WHO Patient Safety Curriculum Guide Multi-professional Edition. Geneva: WHO (2011).

[12] Management Science for Health Ensuring Good Dispensing Practices. Arlington, VA: MSH (2012).

[13] FMHCA US Manual for Medicines Good Dispensing Practice. Addis Ababa: FMHACA (2012). p. 1–76.

[14] Ameh D Wallymahmmed A Mackenzie G Patient knowledge of their dispensed drugs in rural Gambia. Int J Sci Basic Appl Res. (2014) 16:61–85. Available online at: http://gssrr.org/index.php?journal=JournalOfBasicAndApplied

[15] Yabite H Tessema S Wabe NT Dispensed Medications: labeling patterns and patient knowledge at a tertiary care university hospital in Southwest Ethiopia. Ther Innov Regul Sci. (2012) 46:688–93. 10.1177/0092861512456977

[16] Alkatheri AM Albekairy AM. Does the patients' educational level and previous counseling affect their medication knowledge? Ann Thorac Med. (2013) 8:105–8. 10.4103/1817-1737.109823 23741273 PMC3667438

[17] Hussain S Hussain AAS Hussain K Asif MA Khalil MM Abdel Rahman DA Pharmacist–patient counselling in Dubai: assessment and reflection on patient satisfaction. Eur J Hosp Pharm. (2013) 20:241–47. 10.1136/ejhpharm-2012-000263

[18] Marks JR Schectman JM Groninger H Plews-Ogan ML. The association of health literacy and socio-demographic factors with medication knowledge. Patient Educ Couns. (2010) 78:372–6. 10.1016/j.pec.2009.06.017 19773144

[19] Kerzman H Baron-Epel O Toren O. What do discharged patients know about their medication? Patient Educ Couns. (2005) 56:276–82. 10.1016/j.pec.2004.02.019 15721969

[20] Terefe A Chanie T Assessment of patients misunderstanding of dosage regimen instructions among adolescent and adult outpatients in ethiopia: the case of a primary hospital. Int J Pharm Sci Res. (2014) 5:446–53

[21] Watermeyer J Penn C Communicating dosage instructions across cultural and linguistic barriers: pharmacist-patient interactions in a South African antiretroviral clinic. Stellenbosch Papers Linguist Plus (2009) 39:107–25. 10.5842/39-0-77

[22] Ngoh LN. Health literacy: a barrier to pharmacist–patient communication and medication adherence. J Am Pharm Assoc (2009) 49:e132–46. 10.1331/JAPhA.2009.07075 19748861

[23] Phokeo V Hyman I. Provision of pharmaceutical care to patients with limited English proficiency. Am J Health Syst Pharm. (2007) 64:423–9. 10.2146/ajhp060082 17299181

[24] Ngo-Metzger Q Sorkin DH Phillips RS Greenfield S Massagli MP Clarridge B Providing high-quality care for limited English-proficiency patients: the importance of language concordance and interpreter use. J Gen Intern Med. (2007) 22:324–30. 10.1007/s11606-007-0340-z 17957419 PMC2078537

[25] Gonzalvo J Schmelz A Hudmon KS. Community pharmacist and technician communication with Spanish speaking patients: needs assessment. Am Pharm Assoc. (2012) 52:363–6. 10.1331/JAPhA.2012.10153 22618977

[26] Sandra B Sascha R Allison W Danny L Elizabeth M Pharmacist-patient medication communication during admission and discharge in specialty hospital settings: implications for person centered healthcare. Int J Person Center Med. (2014) 4:90–105.

[27] Negri B Brown LD Hernández O Rosenbaum J Roter D Improving interpersonal communication between health care providers and clients. Qual Assur Methodol Refin Ser. (1997) 1–64.

[28] Garjani A Rahbar M Ghafourian T Maleki N Garjani AF Salimnejad M. Relationship of pharmacist interaction with patient knowledge of dispensed drugs and patient satisfaction. East Mediterr Health J. (2009) 15:934–43. 20187545

[29] Enid M Ping-yu C Jie X Betty C Bruce B Nonverbal interpersonal interactions in clinical encounters and patient perceptions of empathy. J Participat Med. (2013) 5:e33.

[30] Hirko N Edessa D. Factors influencing the exit knowledge of patients for dispensed drugs at outpatient pharmacy of Hiwot Fana Specialized University Hospital, eastern ethiopia. Patient Prefer Adherence (2017) 11:205–12. 10.2147/PPA.S128658 28223781 PMC5308573

[31] Fentie M Mekonnen T Tessema M Yeshaw M Ayelign T Atinafu T Assessment of patients' knowledge to their dispensed medications in pharmacies. Int J Pharm Chem Sci (2014) 3:845–50. Available online at: https://www.researchgate.net/publication/298708050

[32] Okumura LM Rotta I Correr CJ. Assessment of pharmacist-led patient counseling in randomized controlled trials: a systematic review. Int J Clin Pharm. (2014) 36:882–91. 10.1007/s11096-014-9982-1 25052621

[33] Kaboli PJ Hoth AB McClimon BJ Schnipper JL. Clinical pharmacists and inpatient medical care: a systematic review. Arch Intern Med (2006) 166:955–64. 10.1001/archinte.166.9.955 16682568

[34] Keshishian F Colodny N Boone RT. Physician–patient and pharmacist–patient communication: geriatrics' perceptions and opinions. Patient Educ Counsel (2008) 71:265–84. 10.1016/j.pec.2008.01.004 18308499

[35] The American Society on Aging (ASA) The American Society of Consultant Pharmacists (ASCP) foundation. Adult Medication: Improving Medication Adherence in Older Adults (2006). 29076448

[36] Perri M Communication skills for pharmacists. Am J Pharm Educ. (2006) 70:1–3. Available online at: https://www.researchgate.net/publication/2540659017136144

[37] Marissa C Salvo MC Cannon-Breland ML Motivational interviewing for medication adherence. Pharm Today (2015) 21:81–9. Available online at: www.pharmacytoday.org 10.1331/JAPhA.2015.15532 26161493

