# Supplementary material for: Exit-Knowledge of Ambulatory Patients About Medications Dispensed in Government Hospital in Eastern Ethiopia: The Need for Focused Patient Counseling
Source: Front Public Health. 2018 Sep 5;6:254. doi: 10.3389/fpubh.2018.00254 (PMC6133987; doi:10.3389/fpubh.2018.00254)
Supplement: Supplementary file 1 [file Table_1.DOCX]

Voluntary informed consent form

Hello, my name is______________________________. I am a clinical pharmacist at Haramaya University. I would like to ask you few questions regarding your knowledge about the medication use and perception about the service provided at the outpatient pharmacy of Federal Harar Police Hospital. The interview would take 10-15 minutes of your time. The purpose of this study is to assess patient knowledge and perception with pharmaceutical services provided in this hospital. This will be helpful in improving the quality of the health services in general and the pharmaceutical care in particular. Your participation is completely voluntary. You can refuse to answer any questions and/or withdraw from the study at any time without any problem to you or the services you get in the hospital. All your responses will remain strictly confidential: the hospital staff will not have access to your responses. Your name will not be recorded and your responses will not be linked to your identity at any time. Do I have your permission to continue?

Yes No If yes, put the signature hereunder

Name of the interviewee _____________________

Signature__________________________________

Date of Interview_________________

QUESTIONNAIRE

| PART I: Socio-demographic characteristics and perceived communication and interaction status of patients with dispensers | | | |
| --- | --- | --- | --- |
| S./NO | Questions | Alternative Choice | Code |
| SDC1 | Sex | Male=1  Female =2 |  |
| SDC2 | Age | __________years |  |
| SDC3 | Area of residence | Urban =1  Rural =2  Homeless=3 |  |
| SDC4 | Ethnicity | Oromo=1  Adare=2  Amhara=3  Tigrae=4  Somali=5  Other(specify)=99 |  |
| SDC5 | Religion | Muslim=1  Orthodox =2  Protestant=3  Catholic=4  ---------Other(specify)=99 |  |
| SDC6 | Marital status | Single=1  Married=2  Divorced=3  Windowed=4  Separated=5 |  |
| SDC7 | Educational level | Illiterate=1  Can read and write=.2  Primary school (1-8)=3  Secondary school (9-12^)^=4  Tertiary =5 |  |
| SDC8 | Occupation | Farmer=1  Government employee=2  Merchant=3  Daily laborer=4  Private org. employee=5  Student =6  Other(specify)=99 |  |
| SDC10 | Frequency of Pharmacy visit | First time=1  Second time=2  Repeat visit=3 |  |

| IP1 | Your interaction with the pharmacist | Poor =1  Moderate =2  Good =3 |  |
| --- | --- | --- | --- |
| IP2 | Primary language of communication | Amharic =1  Afan Oromo =2  Adarigna=3  Somali =4  other(specify)=99 |  |
| IP3 | The voice and tone of the pharmacy personnel | Clear =1  Not clear=2 |  |
| IP4 | Perception on the comfort and cleanness of waiting area | Very uncomfortable=1  Uncomfortable =2  Neutral=3  Comfortable=4  Very comfortable=5 |  |
| IP5 | The politeness and interest of pharmacy service providers | Very polite =1  Polite=2  Neutral=3  Impolite=4  Very impolite=5 |  |
| IP6 | Clarity of the pharmacy service provider’s instructions on how to take your medication | Clear=1  Neutral=2  Not clear=3 |  |
| IP7 | Perceived sufficiency of dispensers' information | Enough=1  Not enough=2  I don’t know=3 |  |

| PART II: Exit-knowledge status using WHO patient care Indicator | | | |
| --- | --- | --- | --- |
| S./No | Questions (Recalling capability) | Alternative Choice | Code |
| QC1 | Has he/she recalled the name of the medication (s)? | No 1 2 Yes |  |
| QC2 | Indication of each medication | No 1 2 Yes |  |
| QC3 | Each medication’s route of administration | No 1 2 Yes |  |
| QC4 | The duration of treatment for each medication | No 1 2 Yes |  |
| QC5 | The frequency of administration of each medication | No 1 2 Yes |  |
| QC6 | What to avoid while taking the medication (s) | No 1 2 Yes |  |
| QC7 | The possible side effects of the medication (s) | No 1 2 Yes |  |
| QC8 | Instructions on taking each medication | No 1 2 Yes |  |
| QC9 | Actions to be taken in case of missed doses | No 1 2 Yes |  |
| QC10 | The proper storage of your medication | No 1 2 Yes |  |
| QC11 | Labeling on the medication (s) | No 1 2 Yes |  |
| QC12 | The outcomes he/she expect from his/her medication therapy | No 1 2 Yes |  |

Thank You for Your Time and Cooperation . . . !
